# Longitudinal correlates of quitting e-cigarettes in the United States

**DOI:** 10.1016/j.pmedr.2025.103197

**Published:** 2025-08-06

**Authors:** Mayank Sakhuja, Shayna Farris, Tara Licciardello Queen, Marissa G. Hall, Ebbie Kalan, Paschal Sheeran, Kurt M. Ribisl, Noel T. Brewer

**Affiliations:** a Department of Health Behavior, Gillings School of Global Public Health, University of North Carolina, Chapel Hill, NC, United States; b Lineberger Comprehensive Cancer Center, University of North Carolina, Chapel Hill, NC, United States; c Department of Psychology and Neuroscience, University of North Carolina, Chapel Hill, NC, United States; d Department of Behavioral and Community Health, University of Maryland, College Park, MD, United States

**Keywords:** Vapes, *E*-cigarettes, Quitting vaping, Quitting e-cigarettes, Predictors of quitting

## Abstract

**Objective:**

*E*-cigarette use has risen markedly among young adults, despite efforts to curb this trend. To inform future programs and policies, we sought to identify longitudinal correlates of quitting e-cigarette use in the United States (US).

**Methods:**

The study design was longitudinal. A nationally representative sample of 1138 US adults and adolescents who used e-cigarettes took a baseline online survey between Nov 2022 and Jan 2023. Six months later, 844 respondents completed a follow-up survey. Analyses used weighted simultaneous multivariable logistic regression that included demographic and vaping characteristics assessed at baseline to predict quitting e-cigarettes at 6-month follow-up.

**Results:**

At 6-month follow up, 15 % of respondents had quit e-cigarettes. Quitting was associated with having stronger quit intentions (adjusted odds ratio [aOR] = 1.49, 95 % CI = 1.09, 2.04), occasional use (aOR = 5.93, 95 % CI = 3.11, 11.30), and lower nicotine dependence (aOR = 2.56, 95 % CI = 1.38, 4.76). Quitting was also more common among gay, lesbian, or bisexual respondents than straight respondents (22 % vs. 13 %, aOR = 2.20, 95 % CI = 1.10, 4.38) and females than males (18 % vs. 11 %, OR 1.88; 95 % CI 1.06, 3.34).

**Conclusions:**

Greater motivation to quit and lower markers of nicotine dependence were associated with quitting vaping at 6 months. Interventions focused on reducing nicotine dependence and increasing quit intentions, especially among daily users, could support e-cigarette cessation.

## Highlights

• National study followed US adolescent and adult e-cigarette users for 6 months.
• Lower nicotine dependence predicted higher odds of quitting e-cigarettes.
• Stronger quit intentions were linked to greater likelihood of quitting e-cigarettes.
• Female and lesbian, gay, or bisexual respondents had higher e-cigarette quit rates.
• Daily e-cigarette users were less likely to quit than occasional users.

## 1 Introduction

E-cigarette sales in the United States went up by 46 % between 2020 (15.7 million units) and 2023 (22.9 million units), with flavored products accounting for 80 % of the total sales and rising by 64 % (Centers for Disease Control and Prevention Foundation, 2023). Adult e-cigarette use grew from 4.5 % to 6.5 % between 2019 and 2023 (Centers for Disease Control and Prevention, 2025). Among US adults who used e-cigarettes in 2021, nearly a third (29 %) used both e-cigarettes and cigarettes (Centers for Disease Control and Prevention, 2021). Demographic patterns show that men, young adults, individuals with lower education and incomes, and those who identify as lesbian, gay, or bisexual use e-cigarettes at a higher rate (Cornelius, 2023; Kramarow and Elgaddal, 2023). As of 2024, 6 % (1.6 million) of US middle and high school students reported using e-cigarettes or vapes, with 90 % using flavored products (United States Food and Drug Administration, 2025). About a quarter used e-cigarettes daily, and a third used at least 20 days per month (United States Food and Drug Administration, 2025). Dual use was also common among youth, with one-third of high school and half of middle school students reported using other tobacco products with e-cigarettes (Wang, 2019).

Most e-cigarettes contain nicotine (United States Department of Health and Human Services, 2016), which is highly addictive. Nicotine can harm mental health (Obisesan et al., 2019) and brain development during adolescence (United States Department of Health and Human Services, 2016). Adolescents who use e-cigarettes containing nicotine may face a higher risk of future addiction to cigarettes and other combustible nicotine products (Hammond et al., 2017; Soneji et al., 2017). *E*-cigarette aerosol also contains harmful substances, including cancer-causing chemicals, heavy metals, and flavorings linked to lung disease (United States Department of Health and Human Services, 2016). People who use both e-cigarettes and cigarettes face higher exposure to nicotine and harmful toxicants, leading to worse respiratory outcomes compared to using either product alone (Reddy et al., 2021; Smith et al., 2021). The extent of these harms can vary depending on the intensity and pattern of dual use (Holt et al., 2023; Hartmann-Boyce et al., 2023). With increasing evidence about the health risks of using e-cigarettes, most people who vape express a desire to quit. Findings from a nationally representative cross-sectional survey data showed that 60 % of US adults who used e-cigarettes planned to quit, and 15 % attempted to quit in the last 12 months (Palmer et al., 2021). Similarly, the majority of US middle and high school students who used e-cigarettes wanted to quit, with 64 % expressing a desire to stop, and 67 % having made quit attempts in the last 12 months (Zhang et al., 2022). However, little is known about the correlates of successful quitting over time.

Previous studies have identified correlates of vaping quit attempts, including dual and occasional e-cigarette use, having a friend who vaped, higher tobacco dependence, and higher perceived harm from vaping (Kasza et al., 2020; Cuccia et al., 2021; Kundu et al., 2024). Higher vaping quit intentions have been associated with higher tobacco dependence and perceptions of harm and occasional e-cigarette use (Cuccia et al., 2021; Kundu et al., 2024). Limited research has focused on factors associated with successful quitting over time. Our study sought to bridge this gap by identifying respondent characteristics and baseline vaping behaviors associated with successful e-cigarette quitting six months later.

## 2 Methods

### 2.1 Participants

Respondents were English-speaking US adults (ages 18+ years) and adolescents (ages 13–17 years) who used e-cigarettes somedays or every day and spoke English. Participants were a national probability sample recruited through the Ipsos Knowledge Panel, which covers 97 % of US households. Panelists are selected through random sampling of telephone numbers and residential addresses and invited to join via telephone or mail. Once enrolled, they receive unique log-in credentials and regular email invitations to participate in research studies. Of the 1138 respondents to the baseline survey, 844 completed the follow-up survey. They are the analytic sample for our study.

### 2.2 Procedures

This was a longitudinal study with data collection at baseline and 6 month follow up. Respondents completed the baseline survey between November 2022 and January 2023 and the follow-up survey 6 months later. These online surveys were self-administered. Adults provided consent, and adolescents' parents provided assent for their adolescent children before completing the survey. Respondents received a $10 incentive for participation, an amount set by the survey company. The institutional review board at the University of North Carolina approved the study.

### 2.3 Measures

#### 2.3.1 Outcome assessed at 6-month follow-up

The primary outcome whether respondents had quit e-cigarettes was measured at 6-month follow-up. The question read, “Do you now vape…” and had 3 response options – everyday, somedays, or not at all. We categorized “not at all” as having quit e-cigarettes and other responses as not having quit. This definition reflects behavioral cessation regardless of intent, as we did not assess reasons for stopping.

#### 2.3.2 Covariates assessed at baseline

The baseline survey assessed respondent age, gender (male, female, and non-binary or another gender) (missing, *n* = 1), sexual orientation (straight, gay or lesbian, bisexual, and another sexual orientation) (missing, *n* = 4), race and ethnicity (non-Hispanic White, non-Hispanic Black, non-Hispanic other, and Hispanic) (missing, *n* = 2), education (less than high school, high school or GED, some college or associate degree, and bachelor's degree or higher), and annual household income (less than $24,999, $25,000-49,999, $50,000-74,999, and $75,000 or more in USD) (missing, *n* = 12). We classified respondents as living in poverty (missing, *n* = 32) if their income was at or below 200 % of the 2022 federal poverty level. The baseline survey assessed vaping characteristics including the vape device type (mostly open system, mostly closed system, and both equally) (missing, *n* = 5), vape flavors (no flavor, one flavor, and multiple flavors) (missing, *n* = 2), current dual use of vapes (some days or every day) and cigarettes (some days or every day), vape nicotine concentration (0 mg; 3 or 6 mg; 12, 18, or 24 mg; 30 mg or more; and not sure) (missing, *n* = 2), and vaping frequency (categorized as vaping some days or every day). The baseline survey also assessed nicotine dependence (missing, *n* = 8) using a single item: “How soon after you wake up do you first vape?” We dichotomized responses to reflect vaping within 30 min of waking (nicotine dependent) versus 31 min or later (not dependent).

The baseline survey assessed perceptions and consequences of uncontrolled vaping (Kalan et al., under review). We defined uncontrolled vaping as vaping more than the user prefers. Perceptions and consequences of uncontrolled vaping are conceptually related to nicotine dependence, reflecting self-awareness of problematic patterns or observable outcomes of vaping too much (Whitesell et al., 2024). We included these measures as exploratory constructs. Three items assessed perceptions of uncontrolled vaping: “How often do you find yourself vaping more than you mean to”, “How often do you think that you vape too much”, and “How often do you feel you've lost control over vaping”. Three other items assessed consequences of uncontrolled vaping: “How often do you vape so much you feel sick or queasy”, “How often do you get in trouble at work or school for vaping”, and “How often does needing to vape make you miss out on things”. The 5-point response scale ranged from “Never” (coded as 1) to “Always”. To create composite measures, we took the average of the 3 items for each scale (uncontrolled vaping perceptions α = 0.89, uncontrolled vaping consequences α = 0.70).

The baseline survey assessed whether respondents were vaping while completing the survey (yes, no) (missing, *n* = 1), and their motivation to vape less than their current level, assessed with the question “How interested are you in vaping less than you do now?” Vaping while completing the survey was an exploratory behavioral indicator of dependence. The survey assessed intentions to quit vaping in the next 6 months using three items: “How interested are you in quitting vaping in the next 6 months?”, “How much do you plan to quit vaping in the next 6 months?”, and “How likely are you to quit vaping in the next 6 months?” (Klein et al., 2009). To create a quit intentions scale, we averaged the items yielding a reliable scale (α = 0.87) that ranged from 1 to 5, with higher scores indicating stronger intentions to quit. Finally, the survey assessed perceived harm of using e-cigarettes was measured by two items: “How harmful to your health do you think vaping is?” (missing, *n* = 4) and “Do you think vaping is less harmful, about the same, or more harmful than cigarette smoking?” (missing, *n* = 3) (Population Assessment of Tobacco and Health, 2014).

### 2.4 Statistical analysis

We report unweighted frequencies and weighted means, percentages, odds ratios, and 95 % confidence intervals. To characterize the correlates of quitting e-cigarettes, we used weighted logistic regression. The outcome was quitting e-cigarettes by follow-up, and the predictors were baseline respondent and vaping characteristics. We first conducted bivariate analyses, and we then conducted a multivariable analysis that included statistically significant predictors from the bivariate analyses. Inferential analyses used two-tailed statistical tests with a critical alpha of 0.05.

Weights were constructed using ranking against geodemographic distributions of adolescent and adult e-cigarette use. Benchmarks for post-stratification weights for adolescents were obtained from the 2020 wave of the National Survey on Drug Use and Health and for adults from the 2021 wave of the National Health Interview Survey. Weights for adolescents and adults were combined with adjustments to reflect total e-cigarette users. We present unweighted frequencies and weighted percentages and measures of association calculated using SAS (v 9.4).

## 3 Results

Most respondents were adults (92 %), straight (82 %), and non-Hispanic White (73 %) (Table 1). More than half (54 %) reported using mostly a closed-system vape device and half (50 %) vaped multiple flavors. More than half (59 %) used e-cigarettes every day. About two-thirds (64 %) currently used e-cigarettes only (and not cigarettes), and 36 % used both cigarettes and e-cigarettes. Most (61 %) perceived e-cigarettes to be less harmful than cigarettes. Participants who completed the follow-up survey were older and less likely to be living in poverty (both *p* < .05), but they did not differ on race and ethnicity or nicotine dependence.

**Table 1:** Baseline socio-demographic and vaping characteristics of study participants (US adolescents and adults in winter 2022–23).

	*n*	Weighted %
**RESPONDENT CHARACTERISTICS**		
**Age**, years		
13–17	82	8.5
18–29	207	38.5
30–44	235	31.0
45–59	222	15.9
60+	98	6.0
**Gender**		
Male	334	52.4
Female	492	45.4
Non-binary or another gender	17	2.1
**Sexual orientation**		
Straight	689	81.9
Gay, lesbian, or bisexual	136	16.1
Another orientation	15	1.9
**Race and ethnicity**		
Non-Hispanic, White	618	73.3
Non-Hispanic, Black	60	7.1
Non-Hispanic, Other	103	12.0
Hispanic	61	7.6
**Education**		
Less than high school	61	9.0
High school or GED	181	34.0
Some college or associate degree	355	34.4
Bachelor's degree or higher	247	22.4
**Household income, annual**		
$0–24,999	155	13.3
$25,000–49,999	182	25.9
$50,000–74,999	142	19.5
$75,000 or more	271	41.3
**Living in poverty**		
No	379	55.1
Yes	351	44.9
**Region**		
Northeast	128	12.6
Midwest	212	23.9
South	345	40.6
West	159	23.0
**Rurality**		
Urban	684	82.0
Rural	160	18.0

**VAPING CHARACTERISTICS**		
**Device type**		
Mostly open system	289	32.7
Mostly closed system	448	54.0
Both equally	102	13.3
**Vape flavors**		
No flavors	15	1.8
One flavor	455	48.1
Multiple flavors	372	50.1
**Dual use**		
Vaping only	490	63.9
Dual use with cigarettes	272	36.1
**Nicotine concentration**		
0 mg	43	5.0
3 or 6 mg	410	44.2
12, 18, or 24 mg	132	16.9
30 mg or more	54	7.0
Not sure	203	26.8
**Vaping frequency**		
Some days	346	40.6
Every day	498	59.4
**Vaping nicotine dependence**		
Not dependent	410	49.5
Dependent	426	50.5
**Perceived uncontrolled vaping,** Mean (SD)	2.24 (1.01)	
**Observed uncontrolled vaping,** Mean (SD)	1.37 (0.59)	
**Vaping while completing survey**		
No	455	54.4
Yes	388	45.6
**Vaping harmful to health**		
Not at all	53	5.3
A little	248	30.6
Somewhat	343	43.5
Very	130	14.4
Extremely	66	6.2
**Vaping harmful relative to cigarettes**		
Less harmful	521	60.8
About the same	250	31.4
More harmful	43	4.5
Not sure	27	3.2
**Motivation to vape less**		
Not at all	225	25.0
A little bit	240	30.9
Somewhat	165	19.7
Quite a bit	103	13.4
Very much	111	11.1
**Quit intentions, mean (SD)**	2.33 (1.14)	

*Note.* GED = General education development certificate. Living in poverty was defined as 200 % of the Federal Poverty Level for 2022. Household income, living in poverty and dual use variables did not include data from adolescents (*n* = 82).

Correlates of quitting e-cigarettes.

In the bivariate but not multivariable analyses, adolescents were more likely to quit than young adults and adults (30 % vs. 13 %, OR 2.73; 95 % CI 1.54, 4.82) (Table 2). Those who perceived that e-cigarettes were very or extremely harmful to health (vs not at all, a little, or somewhat) (OR 1.86; 95 % CI 1.13, 3.08) and were equally or more harmful than cigarettes (vs less harmful) (OR 1.60; 95 % CI 0.99, 2.60) were more likely to report successfully quitting e-cigarettes. Respondents who reported not vaping while completing the survey (OR 3.06; 95 % CI 1.73, 5.39), and expressed that they were somewhat, quite a bit or very much (vs. not at all or a little bit) interested in vaping less than their current level (OR 2.28; 95 % CI 1.39, 3.74) were also more likely to report successful quitting.

**Table 2:** Correlates of vaping cessation at 6-months follow-up (US adolescents and adults in winter 2022–23).

	*n* who quit/*n* in category (%)	Bivariate *OR* (95 % CI)	Multivariable *OR* (95 % CI)
**PARTICIPANT CHARACTERISTICS**			
**Age group**			
Adolescents	24/82 (29.5)	2.73 (1.54, 4.82)	1.06 (0.55, 2.08)
Young adults and adults	102/762 (13.3)	1.00	1.00
**Gender**			
Male	41/334 (10.7)	1.00	1.00
Female	82/492 (17.8)	1.80 (1.10, 2.95)	1.88 (1.06, 3.34)
Non-binary or another gender	2/17 (25.3)	2.82 (0.49, 16.16)	2.25 (0.45, 11.06)
**Sexual orientation**			
Straight	93/689 (13.2)	1.00	1.00
Gay, lesbian, bisexual, or another orientation	33/151 (22.0)	1.86 (1.06, 3.26)	2.04 (1.02, 4.08)

**VAPING CHARACTERISTICS**			
**Vaping frequency**			
Some days	103/346 (29.7)	9.15 (5.04, 16.62)	5.41 (2.92, 10.02)
Every day	23/498 (4.4)	1.00	1.00
**Vaping nicotine dependence**			
Not dependent	98/410 (24.0)	5.12 (2.95, 8.87)	2.36 (1.27, 4.39)
Dependent	27/426 (5.8)	1.00	1.00
**Vaping while completing the survey**			
No	98/455 (20.4)	3.06 (1.73, 5.39)	1.37 (0.69, 2.72)
Yes	27/388 (7.7)	1.00	1.00
**Perceived harmfulness of vaping**			
Not at all / A little / Somewhat	81/644 (12.9)	1.00	1.00
Very / Extremely	45/196 (21.6)	1.86 (1.13, 3.08)	1.08 (0.54, 2.18)
**Perceived harm of vaping harm relative to cigarettes**			
Less harmful	60/521 (12.2)	1.00	1.00
About the same / More harmful / Not sure	65/320 (18.3)	1.60 (0.99, 2.60)	0.70 (0.36, 1.34)
**Motivation to vape less**			
Not at all / A little bit	47/465 (10.1)	1.00	1.00
Somewhat / Quite a bit / Very much	79/379 (20.4)	2.28 (1.39, 3.74)	1.41 (0.64, 3.12)
**Quit intentions**		1.64 (1.36, 1.97)	1.50 (1.09, 2.06)

*Note.* Table shows odds ratios (OR) and 95 % confidence intervals (CI) from bivariate and multivariable logistic regression analyses. Only variables that were statistically significant (p < .05) in bivariate analyses are included in this table. The multivariable model includes these variables simultaneously and presents the resulting adjusted odds ratios. Full bivariate results, including non-significant predictors (race and ethnicity, education, income, poverty, region rurality, device type, vape flavors, dual use, nicotine concentration, and uncontrolled vaping) are available in the Supplemental Table. GED = General education development certificate, OR = odds ratio, CI = confidence interval. Living in poverty was defined as 200 % of the Federal Poverty Level for 2022.

In multivariable analysis, female respondents were more likely to quit e-cigarettes than male respondents (18 % vs. 11 %, OR 1.88; 95 % CI 1.06, 3.34) (Table 2). Similarly, respondents who were gay, lesbian, bisexual, or another orientation had higher quit rates than straight respondents (22 % vs. 13 %, OR 2.20; 95 % CI 1.10, 4.38). Respondents who occasionally used e-cigarettes (OR 5.93; 95 % CI 3.11, 11.30) and those who were not nicotine dependent (OR 2.56; 95 % CI 1.38, 4.76) were more likely to quit e-cigarettes compared to those who used them daily and were nicotine dependent. Finally, stronger intentions to quit in the next 6 months were associated with a greater likelihood of quitting e-cigarettes (OR 1.49; 95 % CI 1.09, 2.04).

## 4 Discussion

In this nationally representative US study, about one in six individuals who were using e-cigarettes at baseline reported quitting six months later. This rate is consistent with a Population Assessment of Tobacco and Health (PATH) Study that found 1 in 2 people quit vaping over 4 years (Kalan and Brewer, 2023). In our study, quitting was more common among people who had higher intentions to quit vaping, used e-cigarettes occasionally, were not nicotine dependent, were women, and were lesbian, gay, or bisexual (LGB). Understanding the predictors of e-cigarette quitting may help to inform public health interventions.

Respondents with higher intentions to quit in the next 6 months were more likely to quit e-cigarette use. This finding aligns with previous research highlighting the critical role of quit intentions in cessation across various tobacco products (Cengelli et al., 2012; Li et al., 2010; Vallata et al., 2021). Intention to quit reflects an individual's readiness and motivation to change, making it a key target for interventions designed to promote e-cigarette cessation. Strategies such as motivational interviewing (Heckman et al., 2010; Hettema and Hendricks, 2010), goal-setting (Shoesmith et al., 2021), and personalized quit plans (Sheeran et al., 2025) are particularly effective in enhancing quit intentions and supporting individuals in their cessation journey.

Lower dependence – as indicated by occasional use, no nicotine dependence, and not vaping during the survey – were associated with higher quitting rates in our study. This mirrors results from the PATH Study, which found that non-daily users had greater odds of discontinuing e-cigarette use after 1 year, reinforcing the importance of nicotine dependence as a barrier to cessation (Kasza et al., 2020). However, another study that examined transitions in e-cigarette use behavior over four years using the PATH Study data found that adults with high nicotine dependence were more likely to discontinue e-cigarette use than those with lower dependence (Kalan and Brewer, 2023). It is possible that individuals who are more nicotine dependent may struggle to quit in the short term, as evident from our study, but with more time, they may eventually succeed in quitting. Our findings suggest that quitting e-cigarettes may require multiple quit attempts, and higher dependence may present more significant initial barriers. However, over time, individuals might seek more intensive interventions or support that improves chances of quitting.

Quitting was also more common among LGB and female respondents. While LGB individuals have higher rates of e-cigarette use in previous studies (Truth Initiative, 2022), our study found that they quit e-cigarette use more often when compared to straight counterparts. Similarly, LGB participants in a smoking cessation trial were more likely to remain abstinent from vaping than their heterosexual counterparts at a 3-month follow up (Martinez et al., 2023). However, limited literature is available specifically on e-cigarette cessation among LGB people, and more research is needed to understand the drivers behind this finding. The literature on gender differences in e-cigarette quitting has yielded mixed findings (Kasza et al., 2020; Cuccia et al., 2021; Smith et al., 2016). For example, nationally representative studies of e-cigarette users found that women were less likely than men to make a quit attempt (Cuccia et al., 2021). Similarly, a study analyzing data from the first three waves (2013–2016) of the PATH Study found no gender differences in e-cigarette cessation behaviors, such as discontinuing use or attempting to quit (Kasza et al., 2020). Moreover, a review on gender differences in smoking cessation noted that women generally face more difficulty in maintaining long-term abstinence from cigarettes compared to men (Smith et al., 2016). Gender differences in vaping cessation may be influenced by unique characteristics of e-cigarettes, including their appeal, patterns of use, and perceived harm, which may differ from those of combustible cigarettes. Future research should investigate the role of individual, social, and contextual factors in shaping cessation behaviors and explore how interventions can be tailored to address the unique needs and challenges faced by men and women.

Strengths of our study include a nationally representative sample, weighted data to enhance generalizability, and inclusion of broad range of correlates of e-cigarette quitting, including exploratory constructs such as vaping while completing the survey and uncontrolled vaping. Study limitations include that the observational nature of the data limits our ability to infer a causal relationship between the predictors and e-cigarette quitting. To better establish causality, experimental studies are warranted. Second, the relatively small sample of adolescents may have limited our power to detect findings for this age group. Fewer adolescents participated in the survey because the panel had fewer of them available, and their participation required two layers of approval (parental consent and child assent), which may have limited participation. Third, the study included self-reported data for e-cigarette use and quitting which might be subject to recall and response bias, and the lack of chemical verification could lead to inaccuracies in reported quit rates. Lastly, interpreting e-cigarette quitting as a standalone outcome may have some limitations. For individuals who use e-cigarettes to help them quit smoking, quitting e-cigarettes might increase the risk of relapsing into smoking combustible cigarettes, which could offset the potential health benefits of e-cigarette quitting (Klemperer et al., 2023). Because the survey did not assess cigarette smoking at follow-up, we could not examine whether individuals who quit vaping also quit smoking or transitioned to exclusive cigarette use. Future research should assess both vaping and smoking behaviors over time to better characterize patterns of dual use and cessation trajectories.

In summary, this study contributes valuable insights into the longitudinal predictors of e-cigarette quitting, with quitting relatively common among females, LGB people, people who occasionally use vapes, and those with lower nicotine dependence. Clinical and communication interventions focused on reducing nicotine dependence and increasing quit intentions, especially among those who vape daily and with low intentions to quit, could encourage successful e-cigarette cessation. While other factors such as harm perceptions may play a role, future research should explore how interventions can best strengthen quit intentions and support sustained cessation efforts. This will provide a deeper understanding of the mechanisms underlying successful e-cigarette quitting and inform public health strategies aimed at reducing e-cigarette use.

## Funding acknowledgement

Research reported in this publication was supported by NCI and FDA Center for Tobacco Products (CTP) under award number R01CA246606. The content is solely the responsibility of the authors and does not necessarily represent the official views of the NIH or the Food and Drug Administration.

## CRediT authorship contribution statement

**Mayank Sakhuja:** Writing – review & editing, Writing – original draft, Visualization, Methodology, Investigation, Formal analysis, Conceptualization. **Shayna Farris:** Writing – review & editing, Methodology, Conceptualization. **Tara Licciardello Queen:** Writing – review & editing, Validation, Methodology, Data curation, Conceptualization. **Marissa G. Hall:** Writing – review & editing, Methodology, Conceptualization. **Ebbie Kalan:** Writing – review & editing, Methodology, Conceptualization. **Paschal Sheeran:** Writing – review & editing, Methodology, Conceptualization. **Kurt M. Ribisl:** Writing – review & editing, Methodology, Funding acquisition, Conceptualization. **Noel T. Brewer:** Writing – review & editing, Supervision, Project administration, Methodology, Funding acquisition, Conceptualization.

## Declaration of competing interest

The authors declare that they have no known competing financial interests or personal relationships that could have appeared to influence the work reported in this paper.

## Data Availability

Data will be made available on request.
