# Supplementary material for: Longitudinal correlates of quitting e-cigarettes in the United States
Source: Prev Med Rep. 2025 Aug 6;57:103197. doi: 10.1016/j.pmedr.2025.103197 (PMC12357258; doi:10.1016/j.pmedr.2025.103197)
Supplement: Supplementary file 1 — Appendix A: Supplementary Table [file mmc1.docx]

**Supplementary Table. Correlates of vaping cessation at 6-months follow-up (US adolescents and adults in winter 2022-23)**

|  | *n* who quit/*n* in category (%) | Bivariate  *OR* (95% CI) | Multivariable  *OR* (95% CI) |
| --- | --- | --- | --- |
| **PARTICIPANT CHARACTERISTICS** |  |  |  |
| **Age group** |  |  |  |
| Adolescents | 24/82 (29.5) | 2.73 (1.54, 4.82) | 1.06 (0.55, 2.08) |
| Young adults and adults | 102/762 (13.3) | 1.00 | 1.00 |
| **Gender** |  |  |  |
| Male | 41/334 (10.7) | 1.00 | 1.00 |
| Female | 82/492 (17.8) | 1.80 (1.10, 2.95) | 1.88 (1.06, 3.34) |
| Non-binary or another gender | 2/17 (25.3) | 2.82 (0.49, 16.16) | 2.25 (0.45, 11.06) |
| **Sexual orientation** |  |  |  |
| Straight | 93/689 (13.2) | 1.00 | 1.00 |
| Gay, lesbian, bisexual, or another orientation | 33/151 (22.0) | 1.86 (1.06, 3.26) | 2.04 (1.02, 4.08) |
| **Race** |  |  |  |
| Non-Hispanic, White | 91/618 (14.3) | 0.89 (0.53, 1.52) |  |
| Other races and ethnicities | 35/224 (15.7) | 1.00 |  |
| **Education** |  |  |  |
| Less than high school / High school or GED | 39/242 (12.4) | 0.62 (0.34, 1.12) |  |
| Some college or associate degree | 48/355 (15.0) | 0.78 (0.43, 1.39) |  |
| Bachelor's degree or higher | 39/247 (18.5) | 1.00 |  |
| **Household income, annual** |  |  |  |
| $0-24,999 | 22/155 (11.2) | 0.78 (0.39, 1.58) |  |
| $25,000-49,999 | 29/182 (16.3) | 1.21 (0.62, 2.37) |  |
| $50,000-74,999 | 14/142 (10.0) | 0.69 (0.30, 1.59) |  |
| $75,000 or more | 36/271 (13.8) | 1.00 |  |
| **Living in poverty** |  |  |  |
| No | 49/379 (13.1) | 1.00 |  |
| Yes | 47/351 (13.2) | 1.00 (0.57, 1.75) |  |
| **Region** |  |  |  |
| Northeast | 20/128 (15.0) | 1.07 (0.54, 2.11) |  |
| Midwest | 32/212 (13.9) | 0.98 (0.53, 1.79) |  |
| South | 51/345 (14.1) | 1.00 |  |
| West | 23/159 (16.2) | 1.17 (0.60, 2.28) |  |
| **Rurality** |  |  |  |
| Urban | 99/684 (14.1) | 1.00 |  |
| Rural | 27/160 (17.0) | 1.24 (0.70, 2.18) |  |
|  |  |  |  |
| **VAPING CHARACTERISTICS** |  |  |  |
| **Device type** |  |  |  |
| Mostly closed system | 79/448 (16.9) | 1.77 (0.81, 3.86) |  |
| Both equally | 12/102 (10.3) | 1.00 |  |
| Mostly open system | 34/289 (12.7) | 1.27 (0.54, 3.02) |  |
| **Vape flavors** |  |  |  |
| Unflavored | 3/15 (26.5) | 2.70 (0.58, 12.43) |  |
| One flavor | 74/455 (17.3) | 1.56 (0.95, 2.55) |  |
| Multiple flavors | 49/372 (11.8) | 1.00 |  |
| **Dual use** |  |  |  |
| Vape only | 49/490 (12.1) | 1.00 |  |
| Dual use with cigarettes | 53/272 (15.5) | 1.33 (0.77, 2.28) |  |
| **Nicotine concentration** |  |  |  |
| Without nicotine | 9/43 (26.1) | 2.02 (0.71, 5.72) |  |
| With nicotine | 81/596 (13.6) | 0.90 (0.52, 1.55) |  |
| Not sure | 34/203 (14.9) | 1.00 |  |
| **Vaping frequency** |  |  |  |
| Some days | 103/346 (29.7) | 9.15 (5.04, 16.62) | 5.41 (2.92, 10.02) |
| Every day | 23/498 (4.4) | 1.00 | 1.00 |
| **Vaping nicotine dependence** |  |  |  |
| Not dependent | 98/410 (24.0) | 5.12 (2.95, 8.87) | 2.36 (1.27, 4.39) |
| Dependent | 27/426 (5.8) | 1.00 | 1.00 |
| **Vaping while completing the survey** |  |  |  |
| No | 98/455 (20.4) | 3.06 (1.73, 5.39) | 1.37 (0.69, 2.72) |
| Yes | 27/388 (7.7) | 1.00 | 1.00 |
| **Perceived uncontrolled vaping** |  | 0.86 (0.66, 1.12) |  |
| **Observed uncontrolled vaping** |  | 1.14 (0.79, 1.63) |  |
| **Perceived harmfulness of vaping** |  |  |  |
| Not at all / A little / Somewhat | 81/644 (12.9) | 1.00 | 1.00 |
| Very / Extremely | 45/196 (21.6) | 1.86 (1.13, 3.08) | 1.08 (0.54, 2.18) |
| **Perceived harm of vaping harm relative to cigarettes** |  |  |  |
| Less harmful | 60/521 (12.2) | 1.00 | 1.00 |
| About the same / More harmful / Not sure | 65/320 (18.3) | 1.60 (0.99, 2.60) | 0.70 (0.36, 1.34) |
| **Motivation to vape less** |  |  |  |
| Not at all / A little bit | 47/465 (10.1) | 1.00 | 1.00 |
| Somewhat / Quite a bit / Very much | 79/379 (20.4) | 2.28 (1.39, 3.74) | 1.41 (0.64, 3.12) |
| **Quit intentions** |  | 1.64 (1.36, 1.97) | 1.50 (1.09, 2.06) |

*Note.* Table shows odds ratios (OR) and 95% confidence intervals (CI) from bivariate and multivariable logistic regression analyses. The multivariable model includes statistically significant bivariate variables simultaneously and presents the resulting adjusted odds ratios. GED = General education development certificate, OR = odds ratio, CI = confidence interval. Living in poverty was defined as 200% of the Federal Poverty Level for 2022.
